# Mitochondrial oxidative stress, endothelial function and metabolic control in patients with type II diabetes and periodontitis: A randomised controlled clinical trial

**DOI:** 10.1016/j.ijcard.2018.05.019

**Published:** 2018-11-15

**Authors:** Stefano Masi, Marco Orlandi, Mohamed Parkar, Devina Bhowruth, Isabel Kingston, Caitriona O'Rourke, Agostino Virdis, Aroon Hingorani, Steven J. Hurel, Nikolaos Donos, Francesco D'Aiuto, John Deanfield

**Affiliations:** a Department of Clinical and Experimental Medicine, University of Pisa, Italy; b National Centre for Cardiovascular Prevention and Outcomes, Institute of Cardiovascular Sciences, UCL, London, UK; c Periodontology Unit, UCL Eastman Dental Institute, UCL, London, UK; d Cruciform Teaching Facilities Unit, Faculty of Life Sciences, UCL, London, UK; e Biomaterials and Tissue Engineering, University College London, UK; f Institute of Cardiovascular Science, University College London, UK; g Department of Endocrinology, University College London Hospital, London, UK; h Centre for Oral Clinical Research, Institute of Dentistry, Barts & The London School of Medicine & Dentistry, Queen Mary University of London (QMUL), UK

**Keywords:** Mitochondrial oxidative stress, Endothelial function, Diabetes, Inflammation, Periodontitis

## Abstract

**Background:**

Periodontitis (PD) and type 2 diabetes (T2D) are characterized by increased mitochondrial oxidative stress production (mtROS), which has been associated with a greater risk of cardiovascular diseases (CVD). Intensive PD treatment (IPT) can significantly improve endothelial function and metabolic control, although the mechanisms remain unclear. We explored whether, in patients with PD and T2D, changes of mtROS are associated with improvement of endothelial function and metabolic control after IPT.

**Methods:**

51 patients with T2D and PD were enrolled in a single-blind controlled trial and randomised to either intensive (n = 27) or standard (CPT, n = 24) PD treatment. Levels of mtROS in peripheral blood mononuclear cells (PBMC) were measured using a FACS-based assay at baseline and 24 h, 1 week, 2 and 6 months after PD treatment. Inflammatory cytokines, CVD risk factors, metabolic control and endothelial function were assessed at baseline and 6 months after intervention.

**Results:**

After 6 months from PD treatment, the IPT group had lower mtROS (in both the whole PBMC and lymphocytes), circulating levels of HbA1c, glucose, INF-γ, TNF-α (p < 0.05 for all), and improved endothelial function (p < 0.05) compared to the CPT group. There was an association between higher mtROS and lower endothelial function at baseline (r = −0.39; p = 0.01) and, in the IPT group, changes of mtROS were associated with changes of endothelial function (r = 0.41; p < 0.05).

**Conclusions:**

Reduced mtROS is associated with improved endothelial function and accompanied by better metabolic control in patients with T2D and PD. mtROS could represent a novel therapeutic target to prevent CVD in T2D.

## Highlights

• Treatment of periodontitis reduces mitochondrial ROS in type 2 diabetes (T2D).
• This is associated with improved endothelial function and metabolic control.
• Reduction of mitochondrial ROS might reduce cardiovascular risk in patients with T2D.

## 1 Introduction

Chronic inflammatory diseases account for a substantial proportion of the cardiovascular disease (CVD) morbidity and mortality worldwide. Among these, type 2 diabetes (T2D) and periodontitis (PD) are highly prevalent in the general population and closely interconnected, so that people with PD are at higher risk of T2D, and vice-versa [1]. The risk of cardiorenal mortality (ischaemic heart disease and diabetic nephropathy combined) is three times higher when T2D and PD coexist than in people with T2D alone. While inflammation could account for the increased CVD risk of people with T2D and PD, we have shown that circulating levels of common inflammatory markers are unable to explain the significant improvement of endothelial function and metabolic control observed after intensive periodontal treatment [2,3]. This suggests that there might be more specific and responsive pathways associated with the activation of the inflammatory response, which could underpin the relationship between risk of cardiovascular disease and altered metabolic control in people with T2D and PD.

Mitochondria are central regulators of cellular metabolism and major sources of intracellular reactive oxygen species (mtROS). Increased production of mtROS has been described in patients with T2D, PD and CVD [[4], [5], [6], [7]]. Recently, mtROS production has been identified as an early step in the activation of the inflammatory response, stimulating pro-inflammatory cytokines production [8]. Thus, mtROS could act as potential link between vascular damage and impaired glucometabolic control in people with T2D and PD, explaining their association with systemic inflammation, but this has not been investigated. We set up a randomised clinical trial where we tested whether PD treatment can modify mtROS and whether changes of mtROS might relate with the improved endothelial function and metabolic control observed after PD treatment in patients with T2D.

## 2 Methods

### 2.1 Study design

We have analysed the relationship between mtROS, endothelial function and inflammatory cytokines in the context of a large parallel group, single-blind, randomised, controlled trial (ISCRTN 83229304, http://www.isrctn.com/ISRCTN83229304) which evaluated the effect of periodontal therapy on metabolic control in patients with T2D. Recruitment and study flowchart are shown in the supplementary data file (Supplemental Fig. S1).

Our aim was to include at least 50 consecutive patients with T2D and moderate to severe PD recruited into the trial mentioned above to additional analyses of mitochondrial parameters. Participants were recruited among referral to the Eastman Dental Hospital (London, United Kingdom) and two other local hospitals (St Marys' and Ealing, London) between December 2011 and September 2012. Inclusion criteria were: aged >18 years, diagnosis of T2D (according to the WHO criteria [9] and confirmed in specialist secondary care diabetes clinic), a minimum of 15 teeth, ≥20 sites with periodontal pocket depth (PPD) ≥ 5 mm and radiographic bone loss assessment. Exclusion criteria were: pregnancy/lactation, HIV or Hepatitis (B, C), subjects with uncontrolled systemic diseases or neoplasms, chronic antibiotic therapy or requiring antibiotic coverage for dental procedures, chronic treatment with medications known to affect periodontal status (phenytoin, cyclosporine). Following a baseline visit, each participant recruited into the trial was randomly allocated (using a computer-generated table) to receive either intensive (IPT) or control periodontal therapy (CPT). Minimisation was performed in terms of diabetes onset, smoking status, gender and PD severity. Allocation to treatment was concealed in an opaque envelope and revealed to the clinician on the day of treatment. During the trial, T2D was managed in secondary care diabetes clinic by consultant diabetologists according to national guidelines from the National Institute for Health and Care Excellence (NICE) [10]. Blood samples for PBMC isolation and assessment of mtROS were collected at baseline and 1 week, 2 months and 6 months after treatment, while levels of inflammatory cytokines, CV risk factors, soluble markers of endothelial cell activation and endothelial function (assessed by flow-mediated dilation, FMD) were measured at baseline and 6 months after treatment. With the exception of the study dental staff delivering the treatment and performing the clinical examinations, the vascular examiner, the nurses collecting the anthropometric measures and blood samples, the laboratory staff who processed and analysed the blood samples and other staff involved with the data collection/analyses was masked to the group allocation. All patients gave written informed consent. The study was approved by the local ethics committee (Ref 07/H0714/97, Joint UCL/UCLH Committees on Ethics of Human Research, Committee A).

### 2.2 Periodontal examination and therapy

Periodontal data were recorded at baseline and 2 months and 6 months after treatment, including PPD and recession of the gingival margin relative to the cement-enamel junction at six sites per tooth. The presence or absence of supragingival dental plaque and gingival bleeding on probing was also recorded [11]. The depth and numbers of the gingival pockets were measured using a millimetre signed probe that was manually inserted in the area around each tooth. As deepening of the gingival sulcus associated with greater inflammation and loss of connective tissue is suggestive of more severe active disease, the number of gingival pockets >4 mm in depth (number of pockets) is considered a cumulative measure of active gingival inflammation. Oral hygiene instructions were given to all patients. Teeth were extracted if they were deemed unsalvageable. Patients in the IPT group received an intensive periodontal treatment protocol consisting of an initial single session of whole mouth scaling of the root surfaces under local analgesia (no time limits were enforced for completing the session). Two months later, patients underwent additional cleaning of the teeth with a similar protocol. Additional periodontal surgery was performed if there were deeper residual periodontal pockets and had improved dental hygiene (dental plaque scores <20%). CPT patients received supra-gingival scaling and polishing of all dentition at the same time points as the IPT group (at baseline and after 2 months). In both groups, T2D was managed according with clinical guidelines by local specialist diabetes teams, who were unaware of the group assignment throughout the study.

### 2.3 Mitochondrial ROS production

Peripheral blood mononuclear cells (PBMC) were isolated following standard procedures by density gradient centrifugation with Ficoll (Ficoll-Paque PLUS, GE, UK) from an aliquot of heparinised blood collected at each study visit. Mitochondrial oxidative stress production was assessed by flow cytometry using the mitochondrial probe MitoSOX Red (Invitrogen, UK). This is highly selective for mitochondrial superoxide detection and is considered the gold standard probe for fluorescent detection of mtROS in live cells [[12], [13], [14]], including PMBC [12,13]. We used kit instructions and a previously validated protocol to assess mtROS production [15] in PBMC. After isolation, an aliquot of 2 × 10^6^ PBMC were resuspended in 1 ml of phenol red free RPMI and incubated for 20 min at 37 °C, in the dark and in a 5% CO2 incubator with MitoSOX Red reagent (final concentration of 5 μM). After three washing steps, cells were resuspended in warm buffer, florescence was read using CellQuest software version 3.1f (Becton Dickinson) and post-acquisition analysis was performed using FlowJo software (FlowJo LLC, Oregon, USA). Median intensity fluorescence was used to estimate the average amount of the mitochondrial superoxide production.

Mitochondrial superoxide production was assessed in the whole PBMC population as well as in the lymphocyte and monocyte subpopulations separately identified by forward and side scatter characteristics. In each analysis, an unstained sample was used as a control to exclude cell autofluorescence. For each analysis, a minimum of 1000 events in the monocyte gate was used to acquire the FACS data.

### 2.4 Endothelial function

Endothelium-dependent and glyceryl trinitrate (GTN)-induced vasodilatation of the brachial artery at baseline and 6 months follow-up was assessed by means of ultrasound imaging (Acuson XP 128/10, Siemens) with the use of a 7-MHz linear probe and automated vessel diameter measurements (Brachial Tools, version 3.2.6, Medical Imaging Applications), as previously described [16] and validated by our group in previous clinical trials with a similar experimental design [3]. A single examiner, blind to the patient treatment allocation, acquired the images of the brachial artery in the morning.

### 2.5 LPS, inflammatory, metabolic and soluble markers of endothelial activation assays

All assays were performed at baseline and 6 months. Serum and plasma were separated within 1 h of collection and stored at −70 °C for future analysis. We measured the serum levels of several circulating inflammatory and endothelial cell activation/injury markers using Meso scale multiplex assay (kits Human pro-inflammatory 7-plex and Human vascular injury Panel I, respectively). HbA1c and insulin levels were assayed on an automated analyser (Cobas 8000 analyser, Roche Diagnostics Corp). Serum endotoxin activity was determined by the Limulus amoebocyte lysate test kit with a chromogenic substrate (Lonza, Walkersville, MD) on diluted (1:5, vol/vol in endotoxin-free water) samples. Intra- and inter-coefficient of variations for all assays were <5%. To minimise variability, all assays were performed at the end of the study, in duplicates and on the same ELISA plate at both time points by a technician who was blinded to patient treatment allocation. Homoeostasis Model Assessment (HOMA2) scores were subsequently calculated (https://www.dtu.ox.ac.uk/homacalculator/).

### 2.6 Statistical analysis

As there were no data to enable prediction of changes of mitochondrial function after anti-inflammatory treatment, we based the sample size calculation on the expected changes in FMD after dental treatment. We calculated that 44 patients (22 per study arm) provided 90% power (2 sided, p = 0.05) to detect a 2% difference in FMD between groups at 6 months after therapy, using an estimated standard deviation of 1.6 (derived from our previous published data). Thus, we decided to include at least 50 consecutive patients from our larger randomised trial (anticipating a 10% drop out rate).

All laboratory data were entered into a computer database proofed for entry errors and loaded in SPSS ver21. Changes in mitochondrial function were evaluated by analysis of variance for repeated measures between IPT and CPT groups, including data at baseline, day 1, day 7, 2 months and 6 months. A conservative F-test was used to interpret the model using the Greenhouse-Geisser correction to account for compound symmetry violations. Changes in inflammatory biomarkers and vascular function were analysed by analysis of covariance (with baseline values as a covariate in the model). Correlation analyses were performed by Spearman test. Significance was at p < 0.05.

## 3 Results

Between December 2011 and September 2012, 51 patients were included in the study: 27 allocated to the IPT and 24 to the CPT group. All participants completed the trial. There were no major adverse events reported.

### 3.1 Patient characteristics

Baseline characteristics in the CPT and IPT groups were comparable, including age, gender, smoking status, lipid levels, body-mass index, blood glucose levels (Table 1) and medication use (Supplemental Table S1).

**Table 1:** Baseline characteristics of the patients.

Variable (mean ± SD)	CPT (N = 24)	IPT (M = 27)
Age, years	58 ± 11	56 ± 9
BMI, Kg/m^2^	32 ± 5	32 ± 7
Gender, males	10 (53%)	15 (56%)
Smoking, current	1 (5%)	1 (4%)
Systolic BP, mm Hg	134 ± 19	136 ± 18
Diastolic BP, mm Hg	81 ± 11	84 ± 11
HbA1c, % (mmol/mol)	7.7 (61) ± 1.2	7.9 (63) ± 1.4
Total cholesterol, mmol/l	4.3 ± 1.0	4.3 ± 1.1
HDL, mmol/l	1.3 ± 0.4	1.3 ± 0.4
LDL, mmol/l	2.0 ± 0.9	2.3 ± 0.9
FMD, %	4.18 ± 2.28	4.13 ± 2.98
CRP*, mg/l	1.8 (3.1)	2.2 (3.0)
TNF-α*, pg/ml	3.7 (1.8)	4.0 (1.7)
s-Eselectin*, pg/ml	24.8 (20.2)	25.8 (11.0)
s-Pselectin*, pg/ml	118.8 (35.8)	103.1 (30.1)
INF-γ*, pg/ml	1.1 (2.4)	0.9 (1.9)
mtROS (MitoSOX, MFI)	25.4 ± 12.5	23.4 ± 10.5

Values are expressed as means ± SD or *median (interquartile range) for non-normally distributed variables.

CPT = Control periodontal therapy; IPT = Intensive Periodontal Therapy; BP = blood pressure; CRP = C-reactive protein; TNF-α = Tumor Necrosis Factor-α; INF-γ = Interferon-γ; FMD = Flow Medicated Dilation; mtROS = Mitochondrial oxidative stress production.

### 3.2 Periodontal status and LPS

After 6 months, the IPT group had lower scores of dental plaque (absolute difference 27%; 95% CI, 15–40; p < 0.001) and fewer sites with active periodontal inflammation (average difference in the number of pockets between groups 15; 95% CI, 3–26; p = 0.014) compared to the CPT group (Supplemental Table S2). A trend towards an improvement of PPD and gingival recession was also observed, although differences were not statistically significant. The general improvement of the oral health was accompanied by lower circulating levels of LPS in the IPT than the CPT group 6 months from treatment (unadjusted difference of 2.73 EU/ml; 95% CI, 1.15–6.45; p = 0.023) (Supplemental Fig. S2).

### 3.3 Mitochondrial ROS production

**Fig. 1:**
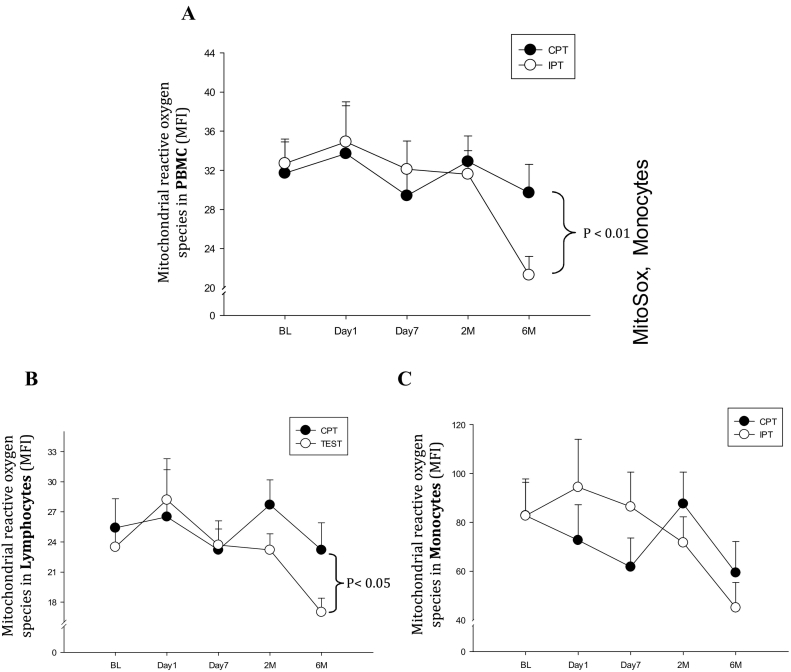
Changes in mitochondrial reactive oxygen species production (mtROS) production during the study period in A) PBMC, B) Lymphocytes, C) Monocytes. I bars represent SE. mtROS production was significantly lower in PBMC (p < 0.01) and lymphocytes (p < 0.05) at 6 months in the IPT compared to the CPT group.

The PBMC of the IPT group had a significantly lower mtROS production compared to the CPT group at 6 months after dental treatment (Fig. 1A). When subpopulations of PBMC were analysed separately, the reduced mtROS generation of PBMC was mainly due to a reduction in oxidative stress production in lymphocytes, although a non-statistically significant trend was also detected in monocytes (Fig. 1B, C).

### 3.4 Vascular and metabolic parameters

**Fig. 2:**
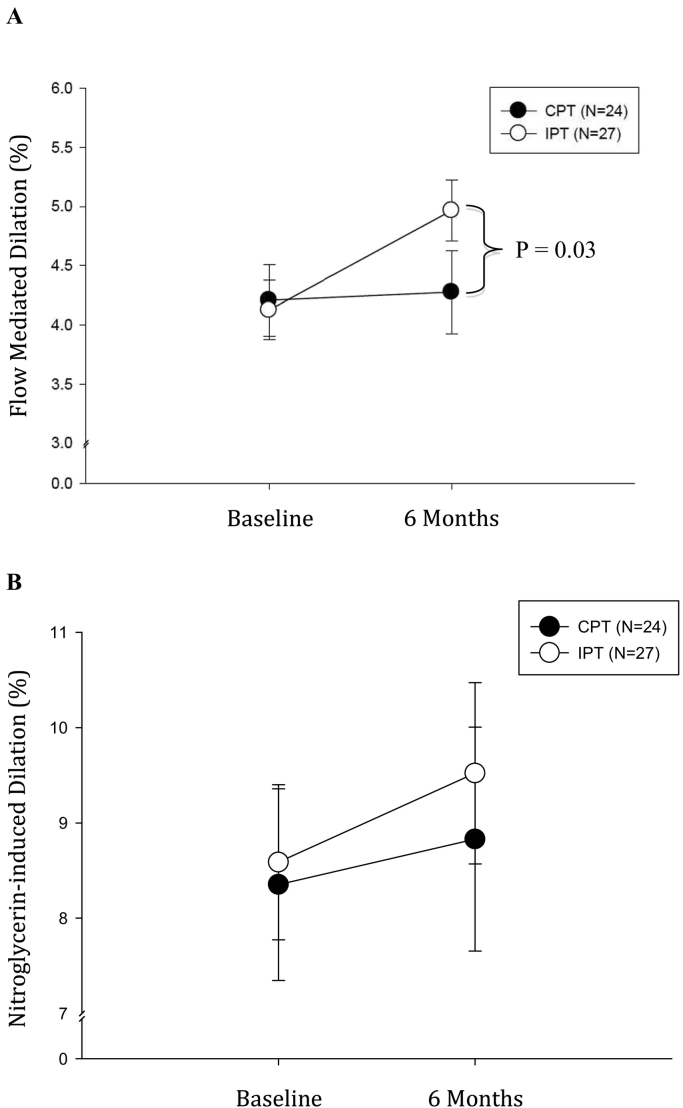
A) Flow-mediated dilatation at baseline and 6 months after periodontal therapy. I bars represent SE. At 6 months of treatment, there was a significant difference in FMD between IPT and CPT groups (p < 0.03). B) Nitroglycerin-dependent dilation of the brachial artery in the IPT and CPT groups. I bars represent SE. At 6 months from treatment, no significant difference of the endothelial independent vasodilation was observed between IPT and CPT groups.

Levels of major cardiovascular risk factors did not differ at baseline and 6 months between IPT and CPT groups and did not change in both groups between baseline and 6 months visits (Supplemental Table S3). There was a significant interaction between treatment and flow-mediated dilatation (p < 0.001). After 6 months, FMD was higher in the IPT than in the CPT group (absolute difference of 0.9%; 95% CI, 0.3–1.4; p = 0.002) (Fig. 2A). Conversely, no changes of GTN-dependent dilatation (endothelial independent) were observed between IPT and CPT groups at 6 months (Fig. 2B).

Patients in the IPT group had lower levels of HbA1c 6 months after therapy compared to CPT patients (average between-group difference of 0.65%, 95%CI 0.22–1.14, p = 0.003). Also, a statistically significant reduction in plasma glucose (average difference between group 1.55 mmol/l, 95%CI 0.25–2.85, p = 0.012) was observed in IPT when compared to CPT patients. No relevant changes were reported in HOMA Index (data not shown).

### 3.5 Markers of inflammation and adhesion

Significant interaction between treatment and time for plasma levels of tumor necrosis factor-α (p = 0.03), interferon-γ (p = 0.04) and soluble E-selectin (p = 0.02) were noted. At 6 months, patients in the IPT group had lower circulating levels of TNF-α (average between groups difference of 0.76 pg/ml, 95% CI, 0.08–1.44, p = 0.02), interferon-γ (average between groups difference of 1.38 pg/ml, 95% CI, 0.26–2.49, p = 0.01) and E–Selectin compared to the CPT group (Supplemental Table S3). No differences were observed in the circulating levels of IL-6 or CRP between groups (Supplemental Table S4).

### 3.6 Relationship between mtROS and other outcomes

**Fig. 3:**
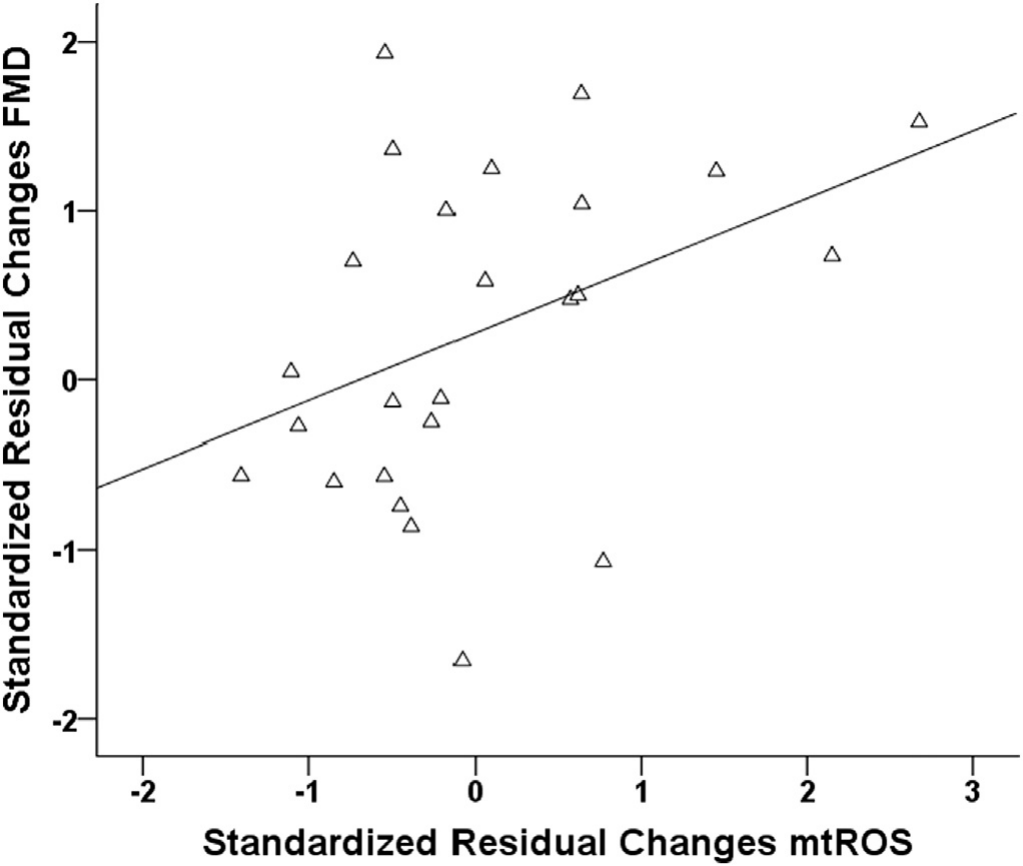
Scatter plot reporting the significant association between changes of mitochondrial reactive oxygen species production (mtROS) in lymphocytes and changes in the flow mediated dilation (FMD) (r = 0.409; p = 0.042).

At baseline, patients with greater lymphocyte mtROS had lower FMD (r = −0.394; p = 0.008). In the IPT group, there was an inverse association between changes in FMD and changes of lymphocyte mtROS, so that the people with a greater recovery of endothelial function also had greater improvement of lymphocyte mtROS (r = 409; p = 0.042) (Fig. 3). Among baseline variables, only lymphocyte mtROS predicted FMD at 6 months (r = −0.368; p = 0.014). In a multivariable regression model including all CVD risk factors (age, BMI, smoking history, gender, systolic blood pressure, total cholesterol, glycaemia, CRP), the association of mtROS with FMD at 6 months remained significant (r = −0.393; p = 0.013). No associations were observed between baseline or 6 months lymphocyte mtROS and both baseline or 6 months levels of fasting plasma glucose, HbA1c, circulating levels of inflammatory markers.

## 4 Discussion

This trial shows, for the first time, that a reduced mtROS production of immuno-inflammatory cells is associated with an improved endothelial function in patients with T2D and PD. Also, an improved mtROS production is accompanied by a significant improvement of the metabolic control in the same study population. These effects were not predicted by circulating levels of common inflammatory markers. Our results suggest mtROS may underpin the relationship between systemic inflammation and both increased risk of CVD and altered metabolic control in patients with type 2 diabetes and PD.

Previous reports have documented elevated mtROS and a dysregulated immune-inflammatory response in patients with PD and T2D [4,7]. Increased production of mtROS is an essential step in the activation of lymphocytes and is a potent trigger of pro-inflammatory cytokine production [8,12,14,17,18]. As activated lymphocytes produce IFN-γ, the reduced intracellular concentration of mtROS detected in these cells might account for the lower circulating levels of this cytokine observed in the IPT group. IFN-γ has an essential role in the regulation of the innate as well as adaptive immunity [[19], [20], [21]]. During viral or bacterial infections, this cytokine is at the top of the inflammatory cascade, as it is produced primarily by activated lymphocytes and can subsequently activate macrophages, resulting in the secretion of other pro-inflammatory mediators (e.g. TNF-α) [22]. In keeping with these functional characteristics, impaired IFN-γ-dependent inflammatory responses are thought to be involved in the pathogenesis of several inflammatory and autoimmune diseases [23]. Conversely, secretion of CRP and IL-6 are late events in the activation of the inflammatory cascade, making their circulating levels more subject to multiple influences and confounding factors. Therefore, our data suggests that mtROS and lower levels of IFN-γ may represent better markers to characterise the dysregulated immune-inflammatory response commonly detected in people with PD and T2D.

We have previously documented that IPT induces a significant improvement of systemic endothelial function at 6 months after PD treatment in patients with and without T2D [2,3]. However, the underlying biological pathways accounting for these results remained unclear, as changes in circulating levels of inflammatory markers were not associated with changes in FMD. We have now shown that mtROS could account for the improved endothelial function observed after IPT. At least two mechanisms could account for these findings. Firstly, inflammatory cytokines could act as transducers between activated inflammatory cells and endothelial cells. For example, IFN-γ is known to induce expression of endothelial cell adhesion molecules, such as vascular cell adhesion molecule-1 (VCAM-1) and intercellular adhesion molecule-1 (ICAM-1). These are involved early in atherogenesis and are crucial for leukocyte recruitment to the plaque [24]. Hernandez-Mijares et al. showed that increased levels of mtROS in patients with diabetes is associated, cross-sectionally, with higher expression of adhesion molecules on endothelial cells, resulting in a higher rolling and adhesion of these cells to the vascular wall [25]. Importantly, Sikorski et al. documented that the expression of cell adhesion molecules on endothelial cells after incubation with IFN-γ can be further intensified by LPS [26]. Based on this previous evidence, the reduced bacteremia achieved by better oral health, combined with reduced mtROS production and levels of IFN-γ following IPT may explain the reduced expression of soluble markers of endothelial cell activation and the improved endothelial function observed in the IPT group at the end of the trial. Secondly, the measure of mtROS in circulating inflammatory cells could reflect a more generalised state of increased mtROS production in other vascular compartments. In patients with T2D, the up-regulation of mtROS production in circulating leukocytes is associated with higher mtROS production in arterioles isolated from subcutaneous fat [27,28]. Consequently, mtROS in circulating leukocytes could have considerable potential as a marker of mtROS in endothelial cells, with the advantage that it can be measured in readily available blood cells.

Together with the reduction of mtROS, the IPT group had a better glycaemic control and lower levels of TNF-α than the CPT group at the end of the trial. Previous meta-analyses have documented associations between PD and risk of T2D or poor glycaemic control [1,29]. Systemic inflammation has been considered a potential mediator of this association, with some cytokines, such as TNF-α, potentially involved in the regulation of systemic insulin-resistance. However, the underlying molecular pathways accounting for the relationship between inflammation and glycaemic control remain unclear. Mitochondria are key regulator of glucose metabolism. An altered balance between nutrients availability and demand for ATP in favour of the former might cause a decrease in the rate of electron flow, that prolongs the lifespan of reactive intermediates at Complexes I and III, ultimately producing a higher amount of mtROS [30]. In turn, an increased mtROS has a primary role in worsening insulin-resistance [[31], [32], [33], [34], [35], [36]], potentially establishing a vicious cycle. As mtROS is also a potent trigger of pro-inflammatory cytokine production, a reduced mtROS induced by the IPT could underpin the relationship between the improved gluco-metabolic control and inflammation observed in our trial.

Our study has several strengths. It is a randomised controlled trial with vascular and laboratory technicians blinded to the patient treatment allocation. Our group was the first to describe, characterise and validate the impact of IPT on endothelial function, markers of inflammation and oxidative stress [3]. We established and validated the use of FMD to measure endothelial function, optimising its reproducibility for large longitudinal studies and clinical trials [3,37]. There are however limitations. The sample size of this study is relatively small. In addition, subjects included in the study where recruited from secondary care specialised diabetic clinics, where patients could have received greater attention and care for the comorbidities commonly associated to T2D (e.g. hypertension and dyslipidaemia). This might have led to the recruitment of a relatively healthy sample of patients affected by T2D, potentially reducing generalisation of our results. Further research is required to confirm our findings in a larger population of patients with diabetes. The FACS analysis is not the most accurate assay to measure mtROS. However, the gold standard techniques used to measure mtROS are laborious and expensive, precluding their use in large human clinical trials. The high specificity and sensitivity of MitoSOX for mitochondrial superoxide production makes this probe the gold-standard method for mtROS assessment by fluorescent staining, and its ability to detect mtROS production in models of T2D and PD has been confirmed recently [6]. To minimise artefacts and optimise our assay, we followed several steps: a) we used cell isolation and staining protocols previously reported and validated, b) we acquired the FACS results immediately after staining and c) we performed gating using the physical characteristics of the cell populations rather than fluorescent-labelled antibodies, as staining with other fluorescent probes is known modify mitochondrial function and influence mtROS. Although these precautions precluded the opportunity to correlate mtROS with markers of lymphocytes/monocytes activation, they reduced several important sources of variability in our results.

## 5 Conclusions

Change of mtROS production following IPT is associated with the improvement of endothelial function and accompanied by a significant improvement of the metabolic control in patients with T2D. mtROS could represent an important and novel therapeutic target to reduce the risk of CV disease and other inflammatory complications in patients with T2D.

## Author contributions

SM, MO, AV, AH, SJH, ND, FDA, JD generated the idea, contributed to draft and to critically reviewed the manuscript; SM, MO, MP, DB, IK, COR acquired the data and critically reviewed the manuscript; MP, DB, IK, COR organized the final dataset; FDA, SM, MO performed the statistical analyses; SM, MO, MP, DB, AV, AH, SJH, ND, FDS, JD interpreted the data.

## Acknowledgements and grant support

We acknowledge the Eastman Clinical Investigation Centre (ECIC) for the constant support provided; Dr. Jeanie Suvan for coordinating the trial, Dr. Nikolaos Gkranias, Dr. Riccardo Zambon, Ms. Maria Currà, Ms. Banbai Hirani, Ms. Tiff Mellor, Ms. Kasia Niziolek for offering the clinical support during the conduct of the trial.

This research was funded by the Diabetes UK (Grant n.: 08/0003594 and 08/0003741) and supported by the National Institute for Health Research (NIHR) University College London Hospitals (UCH) Biomedical Research Centre (BRC) and the Royal College of Surgeons UK. SM was supported by the Rosetrees Trust (Grant n. JS16/M147), UCH-BRC (NIHR), European Society of Hypertension and the British Heart Foundation. MO held a UCL Impact Award partially supported with a fellowship grant from Johnson and Johnson Consumer Services EAME Limited. JD is a British Heart Foundation Professor. FD held a Clinical Senior Lectureship Award supported by the UK Clinical Research Collaboration.

This research project was supported by the National Institute for Health Research University College London Hospitals Biomedical Research Centre. Also, SM was supported by an NIHR Career Development Fellowship from the UCLH/BRC (n. BRC/135/CM/SG – 5982).

## Conflict of interest

None.

## Appendix A Supplementary data

Supplementary material Image 1
