# Supplementary material for: Mitochondrial oxidative stress, endothelial function and metabolic control in patients with type II diabetes and periodontitis: A randomised controlled clinical trial
Source: Int J Cardiol. 2018 Nov 15;271:263–8. doi: 10.1016/j.ijcard.2018.05.019 (PMC6152589; doi:10.1016/j.ijcard.2018.05.019)
Supplement: Supplementary file 1 — Supplementary material [file mmc1.docx]

**ONLINE-ONLY SUPPLEMENTAL MATERIAL**

**MITOCHONDRIAL OXIDATIVE STRESS, ENDOTHELIAL FUNCTION AND METABOLIC CONTROL IN PATIENTS WITH TYPE II DIABETES AND PERIODONTITIS: A RANDOMISED CONTROLLED CLINICAL TRIAL**

**SUPPLEMENTAL TABLES**

**Table S1.** Medication use at baseline in the IPT and CPT groups.

|  | CPT (N=24) | IPT (M=27) |
| --- | --- | --- |
| Biguanides | 20(84.2%) | 26(96.3%) |
| Sulfonylureas | 11(47.4%) | 13(48.1%) |
| Thiazolidinediones Pioglitazone | 4(15.8%) | 1(3.7%) |
| DPP-4 Inhibitors | 5(21.1%) | 4(14.8%) |
| Incretins GLP-1 analogs | 0(0.0%) | 4(14.8%) |
| Betablocker | 8(31.6%) | 6(22.2%) |
| Diuretic | 5(21.1%) | 4(14.8%) |
| Ca channel blocker | 4(15.8%) | 5(18.5%) |
| Alpha blocker | 1(5.3%) | 3(11.1%) |
| Angiotensin-II blocker | 3(10.5%) | 5(18.5%) |
| Ace Inhibitors | 13(52.6%) | 12(44.4%) |
| Statin | 19(78.9%) | 22(81.5%) |
| Aspirin | 11(47.4%) | 7(25.9%) |

**Table S2.** Periodontal parameters at Baseline and 6 Months after Periodontal Therapy

| **Variable** | **Group** | **Baseline** | **6 months** |
| --- | --- | --- | --- |
| Full mouth plaque score (%)^§^ | IPT | 80±13 | 42±20 † |
|  | CPT | 82±15 | 69±20 |
| Full mouth bleeding score (%)^¶^ | IPT | 70±20 | 36±21 † |
|  | CPT | 72±15 | 58±18 |
| Periodontal pocket depth (mm) | IPT | 3.9±0.8 | 2.9±0.7 |
|  | CPT | 3.6±0.7 | 3.3±0.7 |
| Gingival recession (mm) | IPT | 1.2±0.9 | 1.7±1.0 |
|  | CPT | 1.3±0.8 | 1.6±0.8 |
| Number of pockets (n) | IPT | 56±28 | 17±16 † |
|  | CPT | 41±19 | 32±22 |

Values are expressed as means±SD.

† P<0.001 for the comparison with the standard-treatment group.

¶ Scores for full-mouth gingival bleeding were calculated for each patient as the number of sites with gingival bleeding on probing divided by the total number of sites per mouth, multiplied by 100.

§ Scores for full-mouth plaque were calculated for each patient as the number of sites with detectable plaque divided by the total number of sites per mouth, multiplied by 100.

**Supplemental Table S3.** Changes in the levels of common cardiovascular risk factors from baseline to 6 months in both treatment groups.

|  | **CPT** | | | **IPT** | | |
| --- | --- | --- | --- | --- | --- | --- |
|  | **Baseline** | **6 Months** | **P-Values** | **Baseline** | **6 Months** | **P-Values** |
| Systolic Blood Pressure (mmHg) | 134±19 | 136±16 | 0.789 | 136±18 | 133±18 | 0.245 |
| Diastolic Blood Pressure (mmHg) | 81±11 | 78±9 | 0.243 | 84±11 | 82±10 | 0.269 |
| Total Cholesterol Baseline (mmol/l) | 4.3±1.0 | 4.1±1.1 | 0.359 | 4.3±1.1 | 4.2±0.9 | 0.771 |
| HDL-Cholesterol (mmol/l) | 1.3±0.4 | 1.2±0.5 | 0.393 | 1.3±0.4 | 1.4±0.4 | 0.147 |
| LDL-Cholesterol (mmol/l) | 2.0±0.9 | 2.0±0.8 | 0.446 | 2.3±0.9 | 2.2±0.7 | 0.408 |
| Triglycerides (mmol/l) | 2.3±2.6 | 2.2±1.9 | 0.765 | 1.48±1.13 | 1.4±0.9 | 0.563 |
| Creatinine (micromol/l) | 80.6±24.1 | 83.6±30.0 | 0.508 | 76.3±16.1 | 78.6±18.3 | 0.174 |

Differences between baseline and 6 months visit in each group were assessed using paired t-test.

**Table S4.** Circulating biomarkers at baseline and 6 months after treatment

| **Variable** | **Group** | **Baseline** | **6 months** |
| --- | --- | --- | --- |
| CRP, mg/dl* | CPT | 1.80 (3.1) | 2.0 (1.20) |
|  | IPT | 2.20 (3.0) | 2.20 (2.70) |
| IL-6, pg/ml* | CPT | 1.70 (1.70) | 2.27 (2.76) |
|  | IPT | 1.74 (1.48) | 1.66 (1.53) |
| INF-γ, pg/ml* | CPT | 1.1 (2.4) | 2.0 (1.1) |
|  | IPT | 0.9 (1.9) | 0.6 (1.4)†† |
| TNF-α, pg/ml* | CPT | 3.7 (1.8) | 4.1 (5.8) |
|  | IPT | 4.0 (1.7) | 3.7 (3.2)†† |
| s-Eselectin, pg/ml* | CPT | 24.8 (20.2) | 23.2 (8.3) |
|  | IPT | 25.8 (11.0) | 18.1 (13.3)†† |
| s-Pselectin, pg/ml | CPT | 118.8 (35.8) | 104.8 (26.8) |
|  | IPT | 103.1 (30.1) | 82.1 (22.5)†† |

Values are expressed as means±SD or *median (interquartile range) for non-normally distributed variables. †† P<0.05 compared to baseline.

CPT = Control periodontal therapy; IPT = Intensive Periodontal Therapy; TNF-α = Tumor Necrosis Factor-α; INF-γ = Interferon-γ.

**SUPPLEMENTAL FIGURES**

**Figure S1.** Flow chart of the recruitment and study protocol

51 underwent baseline periodontal and endothelial function assessment

Blood samples

PBMC Flow cytometry
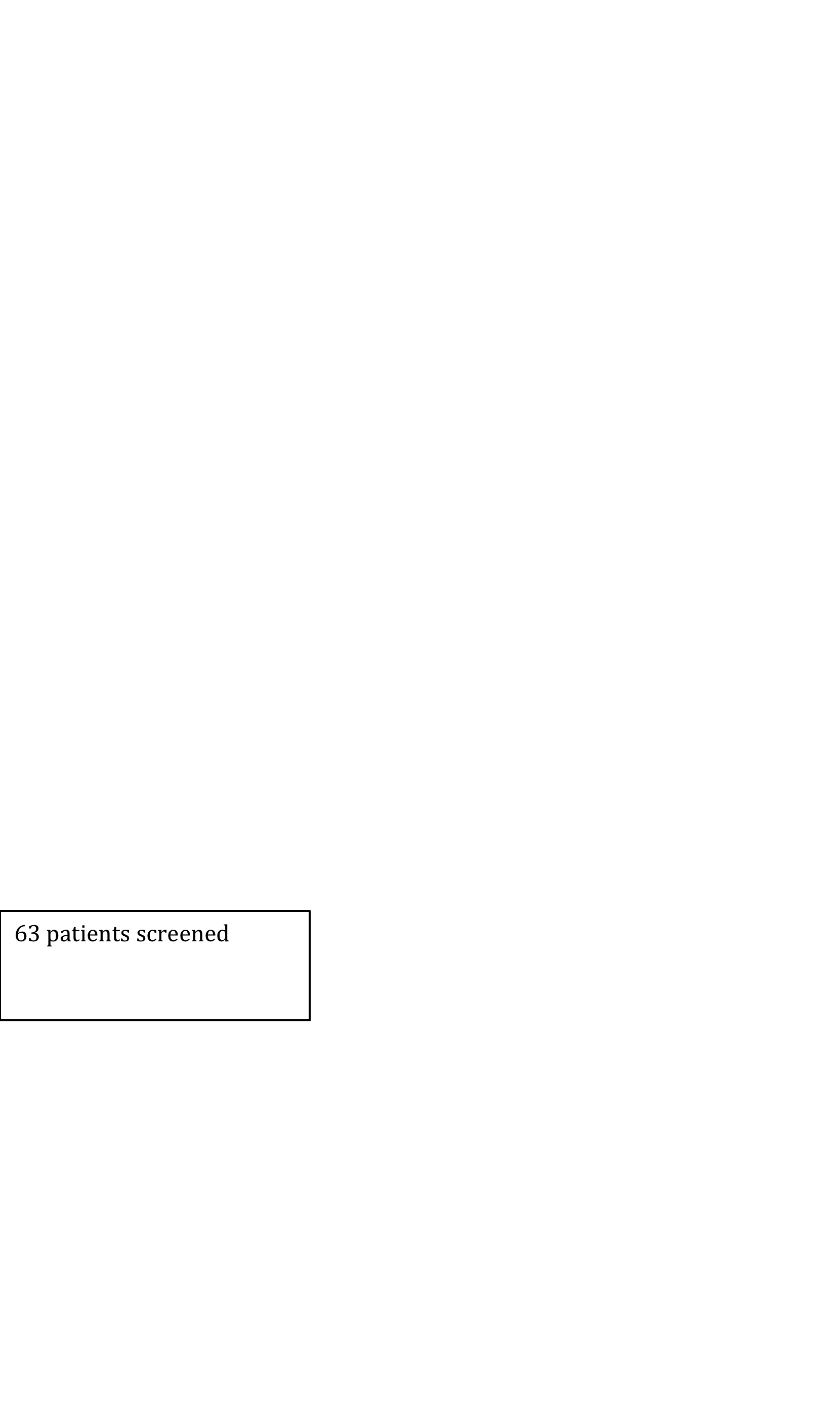
ed

Randomization

24 assigned to CPT

Scaling and polishing

27 assigned to IPT

Scaling and root planing

Day 1 after therapy:

Blood samples

PBMC Flow Cytometry

Day 7 after therapy:

Blood samples

PBMC Flow Cytometry

2 Months after therapy:

Periodontal assessment

Blood samples

PBMC Flow Cytometry

2 Months after therapy:

Scaling and polishing
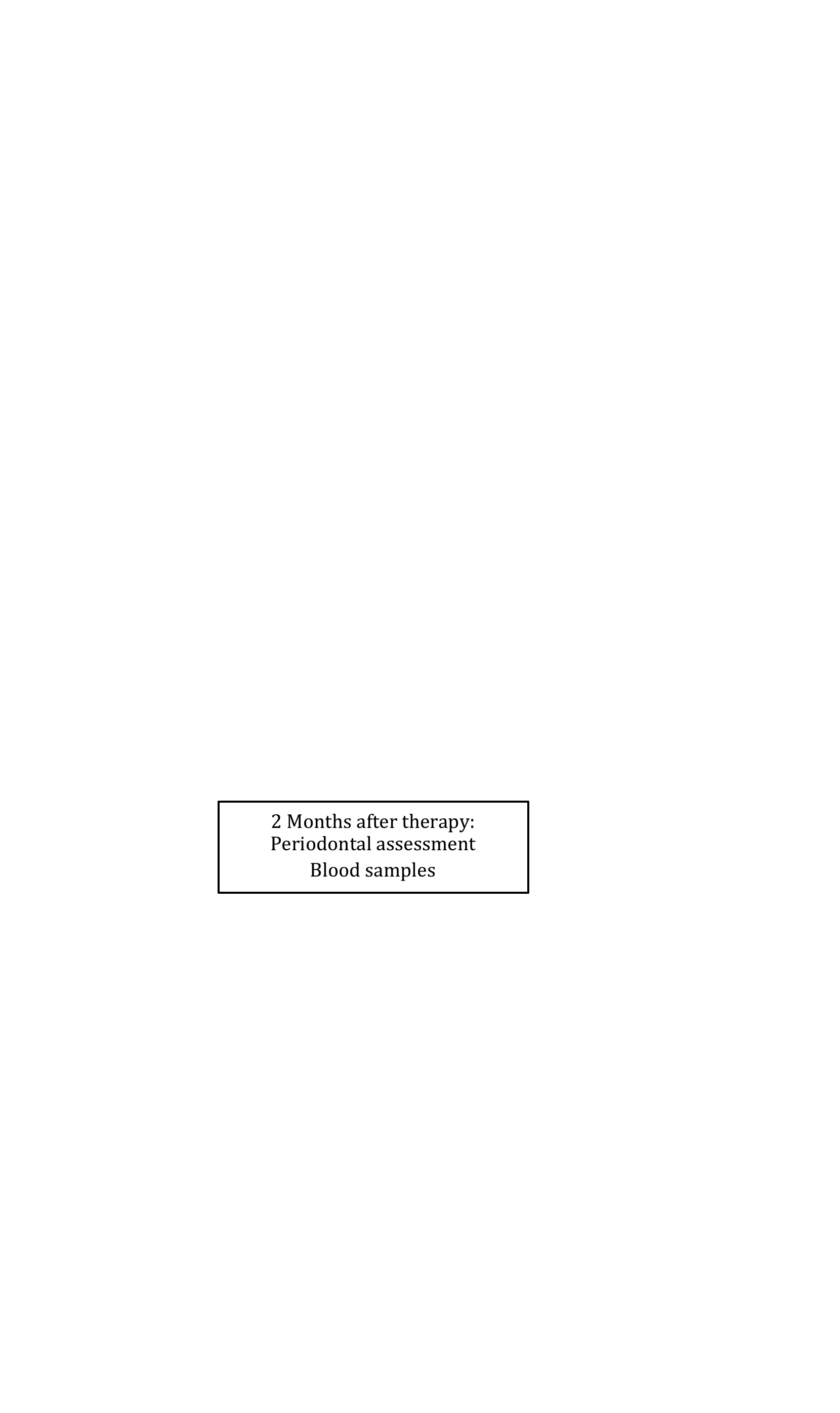


2 Months after therapy:

Modified-Widman Flap/

Scaling and root planing

treatment
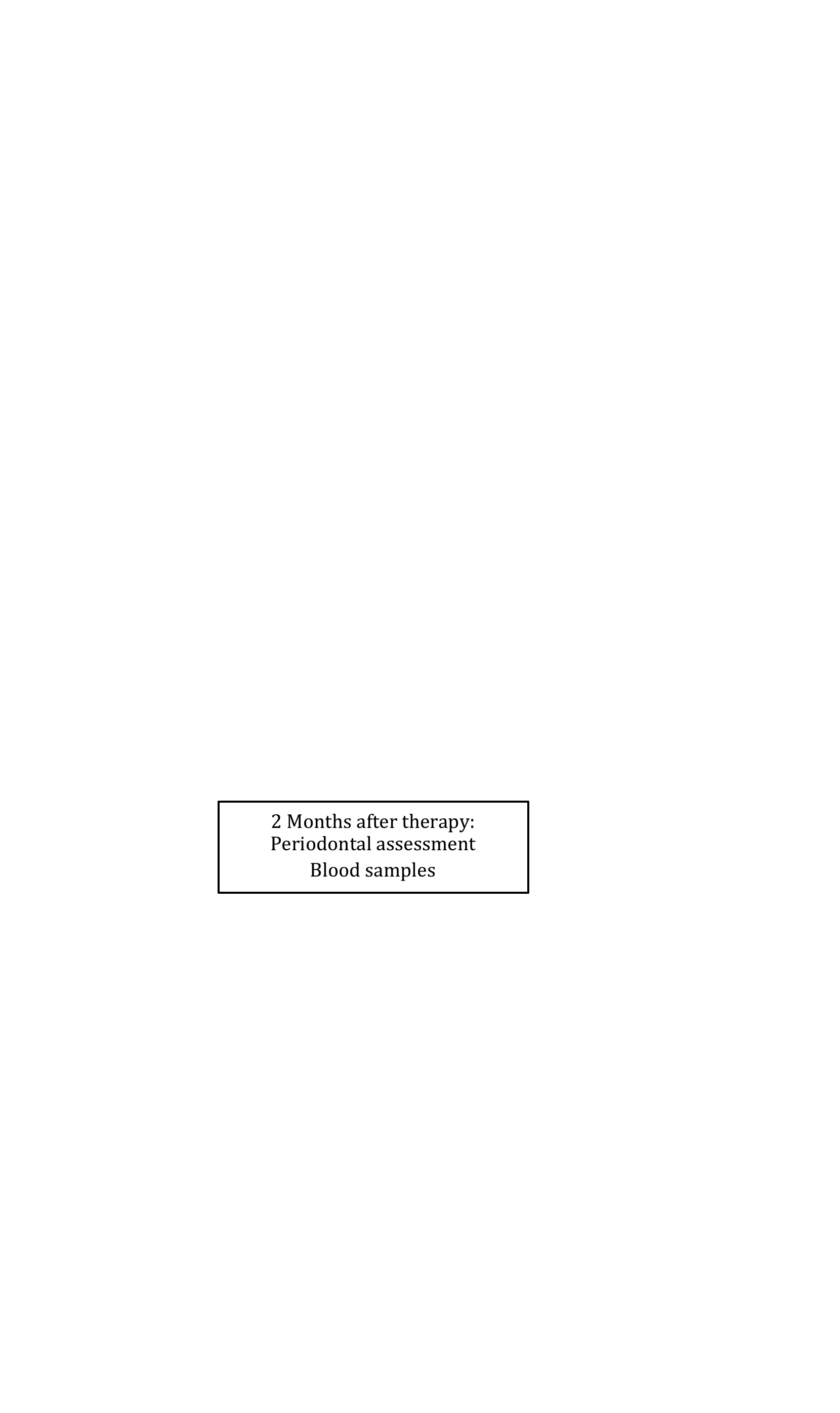


6 Months after therapy:

Periodontal and endothelial function assessment

Blood samples

PBMC Flow Cytometry

24 included in the

intention-to –treat-analysis

27 included in the

intention-to –treat-analysis

**Figure S2.** **Changes in LPS circulating levels in the IPT and CPT groups.** At 6 months, LPS levels were significantly lower in IPT compared to CPT (P<0.05).


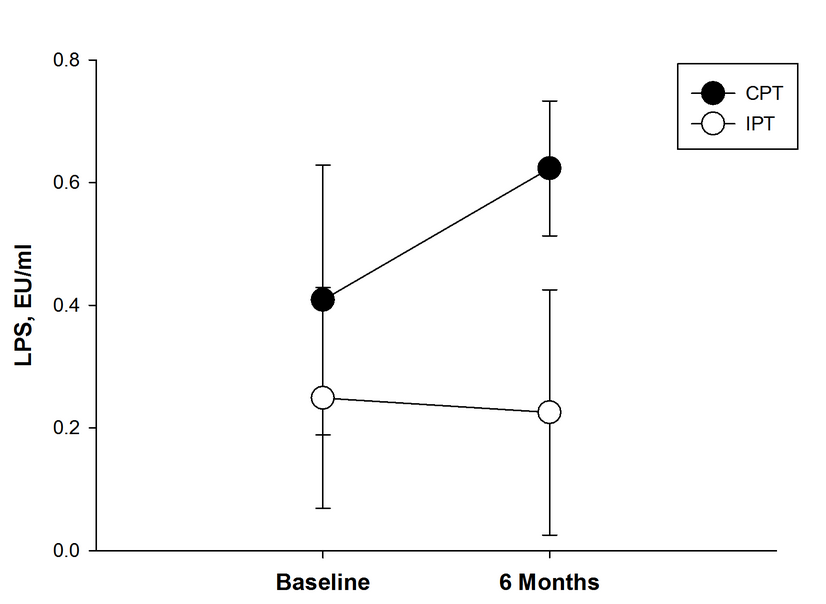


P<0.05
